# Stress granules: emerging players in neurodegenerative diseases

**DOI:** 10.1186/s40035-025-00482-9

**Published:** 2025-05-12

**Authors:** Lin Yuan, Li-Hong Mao, Yong-Ye Huang, Tiago F. Outeiro, Wen Li, Tuane C. R. G. Vieira, Jia-Yi Li

**Affiliations:** 1 Laboratory of Research in Parkinson’s Disease and Related Disorders, Health Sciences Institute, China Medical University, Shenyang, 110122 China; 2 College of Life and Health Sciences, Northeastern University, Shenyang, 110169 China; 3 Department of Experimental Neurodegeneration, Center for Biostructural Imaging of Neurodegeneration, University Medical Center Göttingen, Göttingen, Germany; 4 Max Planck Institute for Multidisciplinary Sciences, Göttingen, Germany; 5 Translational and Clinical Research Institute, Faculty of Medical Sciences, Newcastle University, Newcastle Upon Tyne, UK; 6 Scientific Employee With an Honorary Contract at Deutsches Zentrum Für Neurodegenerative Erkrankungen (DZNE), Göttingen, Germany; 7 Institute of Medical Biochemistry Leopoldo de Meis and National Institute of Science and Technology for Structural Biology and Bioimaging, Federal University of Rio de Janeiro, Rio de Janeiro, RJ 21941-902 Brazil; 8 Neural Plasticity and Repair Unit, Department of Experimental Medical Science Wallenberg Neuroscience Center, BMC, Lund University, 221 84 Lund, Sweden

**Keywords:** Liquid–liquid phase separation, Stress granules, Neurodegenerative disease, RNA-binding protein

## Abstract

Stress granules (SGs) are membraneless organelles formed in the cellular cytoplasm under stressful conditions through liquid–liquid phase separation (LLPS). SG assembly can be both dependent and independent of the eIF2α pathway, whereas cellular protein quality control systems mediate SG disassembly. Chaperones and specific domains of RNA-binding proteins strongly contribute to the regulation SG dynamics. Chronic stress, arising in association with aging, may promote persistent SGs that are difficult to disassemble, thereby acting as a potential pathological nidus for protein aggregation in neurodegenerative diseases (NDDs). In this review, we discuss the dynamics of SGs and the factors involved with SG assembly and disassembly. We also highlight the relationship among LLPS, SGs, and the pathogenesis of different NDDs. More importantly, we summarize SG assembly-disassembly, which may be a double-edged sword in the pathophysiology of NDDs. This review aims to provide new insights into the biology and pathology of LLPS, SGs, and NDDs.

## Introduction

Stress granules (SGs) are ribonucleoprotein (RNP) condensates that are formed in response to cellular stress, such as osmosis, arsenite, heat shock, ultraviolet light (UVC), and viral infections [1, 2]. SGs comprise messenger RNAs (mRNAs) stalled at translation initiation and RNA-binding proteins (RBPs). SGs contribute to minimizing cellular energy demands and maintaining ribostasis and proteostasis by selectively sequestering transcripts and fine-tuning stress responses under physiological conditions. However, the complete functions of SGs are still unclear. A growing body of evidence suggests that dyshomeostasis of SG assembly-disassembly induced by persistent cellular stress is implicated in the pathogenesis of various diseases, such as cancer, neurodegeneration, inflammatory disorders, and viral infections [3–6]. Emerging advances demonstrate that SGs play crucial roles in the pathogenesis of neurodegenerative diseases (NDDs).

As the global population ages, NDDs present significant public health challenges. Protein aggregation and deposition in the central nervous system (CNS) and neuronal loss are major pathological hallmarks of NDDs, suggesting common pathological processes [7, 8]. However, the exact pathogenesis of NDDs remains largely unclear. Proteostasis dysfunction, including the biophysical process of liquid–liquid phase separation (LLPS) of proteins, has been proposed as a contributing factor [9–11]. SGs have been reported to be involved in the pathological processes of NDDs [12]. SGs can accelerate RBP aggregation, resulting in the formation of insoluble inclusions. TDP-43, the pathological aggregate in amyotrophic lateral sclerosis (ALS) and frontotemporal dementia (FTD), mislocalizes from the nucleus to the cytoplasm in response to cellular stress, and disrupts SG disassembly [13, 14], thereby contributing to TDP-43 aggregation [15]. Similarly, multimerization of Ras-GAP SH3 domain-binding protein (G3BP), a pivotal SG nucleator, triggers TDP-43 aggregate formation and aggravate neuronal death [16]. In Alzheimer’s disease (AD), SGs positive for T-cell intracellular antigen-1 (TIA-1) colocalize with tau inclusions [17]. Currently, there are no reports on the relation of SG assembly–disassembly with Parkinson’s disease (PD).

Here, we review SG biogenesis and the dynamics of SG assembly and disassembly in NDDs to address the role of SGs in protein aggregation, with an emphasis on factors influencing SG dynamics. We also discuss the roles of LLPS in the pathology of various NDDs and how RBP domains impact molecular pathogenesis. The aim of this review is to enhance understanding of the molecular mechanisms involved in NDD pathogenesis and to generate novel insights into the pathways involved, thereby illuminating potential therapeutic targets for NDDs.

## Coalescence and composition of SGs

Although LLPS drives SG formation, there are differences in the morphology between liquid droplets observed in cell-free assays and SGs observed in vitro in different cell types [18–20]. SGs in cells exhibit liquid properties, regularly fusing into larger structures [21]. When SGs form after exposure to heat shock, sodium arsenite (SA) or other stress, protein components combine with mRNAs to form SGs ranging from 100 to 1000 nm in diameter [22]. In astrocytes, embryonic fibroblasts, as well as primary cortical and motor neurons, small SGs assemble within minutes upon SA exposure, and coalesce into larger puncta with prolonged exposure [23].

**Fig. 1:**
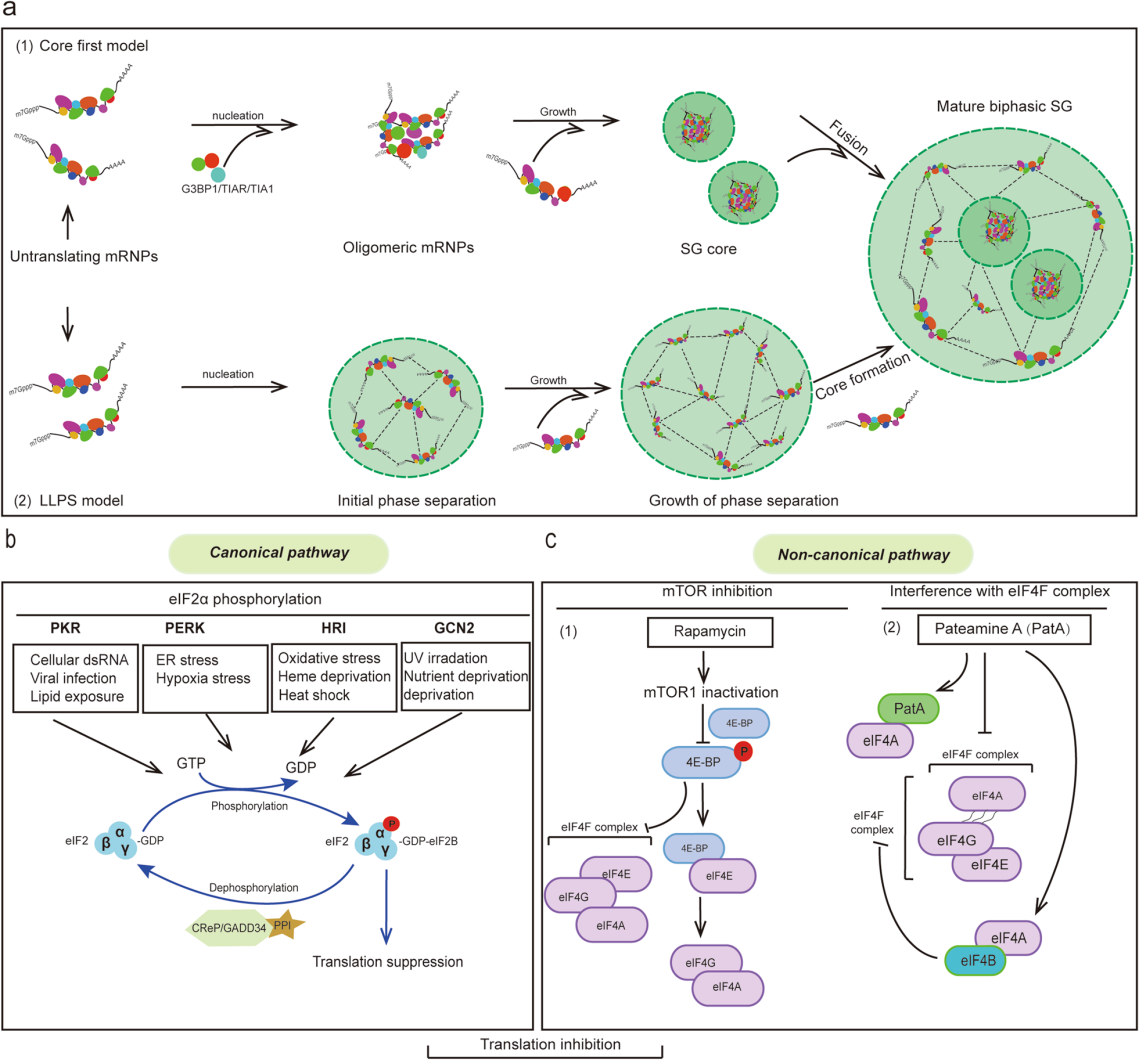
SG assembly pathways. **a** Two models of SG assembly. SG assembly begins with untranslated RNPs in both the “Core First” and “LLPS First” models. (1) “Core First” model: untranslated mRNPs oligomerize into initial oligomeric complexes. G3BP1, TIA1, and TIAR binding drives SG nucleation, recruiting translationally repressed RNPs to form SG cores. These cores fuse and are enveloped by a dynamic "shell" to generate mature biphasic SGs. (2) “LLPS First” model: untranslated mRNPs first undergo phase separation into droplets. Subsequent recruitment of additional mRNPs elevates local protein concentrations, triggering dense core formation within the droplets. **b** The eIF2α phosphorylation-dependent pathway of SG assembly. Cell exposure to diverse stress conditions triggers phosphorylation of eIF2α. The phosphorylation decreases the availability of eIF2-GTP-tRNAi by influencing the interaction of eIF2 with its GTP-GDP exchange factor eIF2B. Dephosphorylation of eIF2α is facilitated by the catalytic subunit of protein phosphatase 1 (PP1), which functions in conjunction with one of two regulatory subunits: GADD34 and CReP. **c** The eIF4F complex, comprising eIF4E, eIF4A, and eIF4G, is a pivotal control point in translation initiation in eukaryotes. eIF4E can bind to 4E-BP to inhibit translation initiation. (1) mTORC1 inactivation induced by rapamycin decreases 4E-BP phosphorylation, and then the eIF4F complex is disrupted by increased formation of the 4E-BP–eIF4E complex. (2) Pateamine A (PatA) is an inhibitor of eukaryotic translation initiation. PatA binds to eIF4A and reduces the interaction between eIF4A and eIF4G, thereby perturbing the function of the eIF4F complex. eIF4A binding with eIF4G is inhibited by the formation of the eIF4A–eIF4B complex, thereby triggering translation inhibition and SG formation

SGs are dynamic structures that exhibit liquid-like behaviors such as flowing, fusing, and component exchanging [20]. SGs consist of a stable “core” substructure surrounded by a dynamic “shell”. The process of the formation of the “double-layer” structure of SGs is still unclear. Currently, there are two models proposed for the assembly of the discrete phases of SG: the “Core First” and the “LLPS First” models [1, 24] (Fig. 1a). In the “Core First” model, the untranslated messenger ribonucleoproteins (mRNPs) oligomerize into core structures first, and then RNPs with weaker interactions are recruited to form the “shell”. These “core” and “shell” structures subsequently coalesce into the mature SG assembly. In the “LLPS First” model, SG formation and maturation precedes core assembly, and is driven by increased concentrations of mRNPs through specific interactions [1]. Super-resolution microscopy has shown that the “core” has more concentrated and less dynamic components than the “shell” layer [25]. Unlike membrane-bound microcompartments, SGs are characterized by rapid shuttling and exchange of components in the “shell” layer with the cytoplasm or processing body (P-body). The heterogeneity of SG proteome components results in different shuttle rates for various components [26]. SGs also coalesce from small to large in response to stress [23].

SGs are structurally characterized by complex networks of protein-RNA interactions. Transiently arrested mRNAs, small ribosomal subunits, translation initiation factors, and RBPs, together with PABP (PolyA-binding protein), G3BP, and T-cell restricted intracellular antigen 1 (TIA1), make up the stalled 48S preinitiation complex, which is a component of SGs [27]. The Mammalian Stress Granules Proteome database highlights that 252 of the 464 SG proteins (54%) are RBPs [28, 29]. Combining proteomic analysis with spatial proteomics, several studies have identified various RBPs as “essential” to SG assembly, including TIA1, G3BP1, G3BP2, and UBAP2L [25, 30–33]. Recently, G3BP1 and G3BP2 have been identified as the central nodes of the core protein-RNA interaction network of SGs in heat-shocked U2OS cells [34]. Additionally, ATP-dependent protein chaperones (such as heat shock protein [Hsp]70 and Hsp40) and multiple RNA and DNA helicases (DEAD-box proteins, MCM, and RVB helicases) are also identified as conserved protein components of SGs, indicating their roles in regulating SG dynamics [25].

In addition, cell type and stress determine approximately 20% of the variability of SG components [28, 30]. The heterogeneities of SG components due to different types of cells and stress should be considered in both physiological processes and pathological conditions.

## Assembly of SGs

SG assembly is driven by inhibition of translation initiation, achieved either independently or via phosphorylation of serine 51 of eukaryotic initiation factor-2α (eIF2α) [22, 35] (Fig. 1b, c). SGs can be induced by increased expression of p-eIF2α, which induces translational arrest, while multivalent interactions mediated by RBPs drive the coalescence of stalled mRNPs into phase-separated condensates [1, 36] Caprin1 facilitates SG assembly by providing additional valency to the multimeric protein-RNA interaction network [37]. Disruption of G3BP1–caprin1 binding leads to reduced G3BP1 condensation with RNA, resulting in reduced SG assembly in HeLa and U2OS cells [38].

SGs can also assemble independently of the p-eIF2α pathway. For example, H_2_O_2_-induced SG assembly is independent of p-eIF2α, but requires remodeling of the eIF4F complex (composed of eIF4E, eIF4G, and eIF4A) responsible for canonical translation initiation [39]. H_2_O_2_ displaces eIF4G and eIF4A from eIF4E while promoting eIF4E interaction with eIF4E-binding protein 1 (4E-BP1), thereby blocking translation initiation and inducing SG assembly. Overexpression of the UBQLN2-P497H mutant reduces SG assembly by inhibiting 4E-BP1 phosphorylation independent of p-eIF2α [40]. Pateamine A can also induce SG assembly in mammalian cells independent of p-eIF2α [41]. Pateamine A inhibits translation initiation by interacting with eIF4A, disrupting ATPase and RNA helicase activity [42]. In yeast and mammals, SG assembly can also be induced under cold shock through parallel pathways, including AMPK (AMP-activated protein kinase) activation, TORC1 inhibition, and PERK (PKR-like ER kinase)-dependent p-eIF2α phosphorylation [43].

## Disassembly of SGs

Maintaining the homeostasis of SG assembly-disassembly is crucial for overall physiological stability. Typically, SGs disassemble when stress is relieved [43, 44], leading to the recovery of mRNA translation and the equilibrium between assembly and disassembly. In both yeast and mammals, SG disassembly occurs in a step-by-step process. Usually, the less concentrated “shell” dissolves first due to weak interactions, followed by dissipation of the internal “core”, which is composed of a stable amyloid-like structure [45].

SG disassembly depends on the type, severity, and duration of external stress. Decreased SG disassembly may cause disequilibrium of SG assembly and disassembly, possibly resulting in pathogenic protein aggregation associated with NDDs [46, 47]. Consistently, cultured neurons derived from ALS patients exposed to chronic oxidative stress display SGs that are more resistant to disassembly, resulting in proteostasis imbalance [46]. The collapse of proteostasis may induce pathological protein aggregation, creating a vicious cycle of "proteostasis imbalance–protein aggregation". Proteome integrity and proteostasis are maintained by protein quality control systems, including the ubiquitin–proteasome system (UPS) and the autophagy-lysosome system (ALP) [48]. Autophagy can directly regulate SG disassembly by modulating G3BP1 ubiquitination via SQSTM1/p62 and CALCOCO2/NDP52 [49]. In NDDs, endogenous and environmental stress causes disturbance of SG disassembly, altering the physiological function of these proteins and leading to protein aggregation and proteotoxic stress, resulting in irreversible neuronal damage. Importantly, impaired SG disassembly has been linked to the aggregation of several proteins associated with NDDs, such as TDP-43, fuse in sarcoma (FUS), and tau [50].

## Factors involved in the regulation of SG assembly and disassembly

### Molecular chaperones regulate SG dynamics

Molecular chaperones regulate protein homeostasis by assisting in protein folding, refolding misfolded proteins, and modulating protein assembly. Growing evidence shows that chaperones are crucial determinants of SG assembly and disassembly. Heat shock proteins Hsp40, Hsp70, and HSPB8 (heat shock protein family B member 8) can be recruited into SGs where specific aggregation-prone protein sequences are highly concentrated, and prevent SGs from progressing into solid aggregation by maintaining their dynamics [51, 52]. For example, Hdj1 (DNAJ heat shock protein family (Hsp40) member B1), a class II Hsp40 protein, can be incorporated into SGs and stabilize the liquid phase of FUS against protein aggregation by regulating SG dynamics [51]. TDP-43 condensates selectively recruit heat shock protein family B (Small) member 1 (HSPB1) upon stress, inhibiting TPD-43 assembly into fibrils. The combined activities of HSPB1, BAG2 (BAG cochaperone 2), and HSPA1 A (heat shock protein family A (Hsp70) member 1 A) facilitate the disassembly of TDP-43 condensates within stressed cells [53]. Hsp90 is required for SG disassembly by binding to DYRK3 (dual-specificity tyrosine-phosphorylation-regulated kinase 3) [54]. Additionally, the yeast Hsp104p mediates the disassembly of solid-like SGs under heat-induced conditions and glucose starvation [55]. Thus, exploring the molecular mechanisms through which chaperones maintain protein homeostasis is crucial for maintaining SG dynamics.

### Energy metabolism modulates SG homeostasis

Changes in cellular energy metabolism can be sources of endogenous stress inducing SG assembly, although the exact mechanisms are not fully understood. Chronic glucose starvation and inhibition of glycolysis induce formation of a unique type of SGs, termed “energy deficiency-induced stress granules” (eSGs) in cells, while moderate ATP reduction delays SG clearance without triggering eSG formation [56]. Acute oxidative stress-induced SG assembly can be inhibited by 2-deoxyglucose combined with CCCP (carbonyl cyanide m-chlorophenyl hydrazone) that block the glycolytic pathway and oxidative phosphorylation, respectively to deplete ATP, suggesting that ATP is required for SG formation [25]. Interestingly, under physiological conditions, ATP binds to TDP-43 arginine residues at a particular molar ratio to dissolve LLPS of TDP-43 [57]. Low concentrations of ATP cause TDP-43 LLPS, while high concentrations dissolve it in vitro [57]. These results demonstrate that intracellular energy is crucial in regulating LLPS, especially for SG assembly. Neurons in the CNS are highly demanding for ATP to maintain action potentials, neuronal function, and synaptic integration [58]. ATP deficiency not only directly affects neurons, but may also contribute to pathological protein aggregation associated with NDDs.

### RNA modulation of SG homeostasis

As scaffolds to initiate RBP nucleation, RNAs can be detected in SGs and are required for SG assembly [59, 60]. RNAs drive SG assembly through RNA-RNA/RNA–protein interactions [61]. SGs are rich in lncRNA NEAT1, which drives TDP-43 assembly to form SGs [62]. SGs are also rich in N6-methyladenosine (m6A)-modified RNA and its binding proteins. The m6A-modified RNA serves as a scaffold to recruit YTHDF2, a m6A reader, to initiate phase separation. In addition, RNA concentration may affect the level of phase separation. Low RNA concentration triggers LLPS via interaction between the positive charges of protein and negative charges of RNA. However, a high concentration of RNA leads to abundance of negative charges, and the repulsion between the charges causes LLPS dissociation [63, 64]. This phenomenon has also been documented for FUS. RNA at a low concentration promotes FUS phase separation, while gradually increasing RNA concentration leads to inhibition of the formation of FUS droplets [64]. Depleting RNA reduces the DEAD-Box helicase 6 (DDX6) assembly capability, suggesting that RNA availability is essential to promote DDX6 granule assembly [65]. RNA length and structure are also critical regulators of phase separation [34, 64]. RNA length > 250 nucleotides and single-strandedness are the basis for RNA ability to promote LLPS with G3BP1 [34]. In addition, restriction of intermolecular RNA-RNA interaction significantly reduces the ability of RNA to induce LLPS of G3BP [34]. However, excessive or high-affinity RNAs may also prevent phase separation by competitively inhibiting protein–protein interactions. Therefore, RNA can either trigger or inhibit LLPS, depending on the context.

### Post-translational modifications (PTMs) and SG homeostasis

PTMs can regulate protein–protein interactions, affecting SG dynamics. Phosphorylation, methylation, and ubiquitination of SG-associated proteins are primary regulators of SG dynamic homeostasis. Phosphorylation regulates phase separation, particularly SG assembly [66, 67]. For example, eIF2α phosphorylation mediates SG assembly as part of the integrated stress response. Phosphorylation of G3BP1 on serine 149 inhibits SG formation and triggers disassembly [68, 69]. Other RBP phosphorylation also impact SG dynamics, with unknown mechanisms. Recent studies have linked methylation to SG assembly regulation. Methylation of G3BP1 arginine residues hinders large SG assembly, while demethylation promotes SG formation [70]. UBAP2L knockdown suppresses SG formation, while its overexpression enhances SG assembly, both effects being modulated by arginine methylation of UBAP2L [71]. FUS mutation resulting in a loss of arginine methylation promotes phase separation and FUS incorporation into SGs [72], potentially contributing to ALS and FTD pathology.

Ubiquitination is involved in different types of stress. As for heat stress, K63 G3BP1 polyubiquitination mediates SG disassembly during recovery from heat stress through the ubiquitin-selective segregase p97/VCP (valosin-containing protein). Ubiquitination is necessary for recovery of cellular activities and SG disassembly but not for SG assembly [73, 74]. The tripartite motif containing 21 (TRIM21), an E3 ubiquitin ligase, has been identified as a central regulator of SG homeostasis and is highly enriched in SGs upon SA treatment. *TRIM21* knockdown decreases G3BP1 ubiquitination and promotes SG assembly, while *TRIM21* overexpression increases G2BP1 ubiquitination and inhibits SG assembly [49]. The ALS pathology-associated SG disassembly is mediated by G3BP1 ubiquitination and its association with autophagy receptors [49], underscoring the regulatory role of ubiquitination in SG dynamics.

Other PTMs, including SUMOylation [75], acetylation [50, 76], O-glycosylation [77], and PARylation (poly (ADP-ribosyl)ation) [78], also regulate SG assembly and disassembly. FUS glutathionylation promotes phase separation, inducing FUS aggregation, indicating that glutathionylation plays a role in the regulation of LLPS [79]. In summary, PTMs significantly influence phase separation dynamics, enabling SGs to respond dynamically and quickly to different stimuli.

## Relationship between LLPS, SGs and pathogenesis of NDDs

Multiple lines of evidence suggest that the SG lifetime can determine the cell fate in NDDs [19, 23, 80]. The sequestration of aggregation-prone proteins within these membraneless compartments significantly impedes SG disassembly kinetics [81]. Although SGs can be induced by various types of stress in eukaryotic cells, most experiments related to SGs in vitro were performed under cell-free conditions due to the lack of methods for monitoring SG dynamics in vivo*.* The conditions leading to LLPS in vitro generally differ from physiological conditions in macromolecule concentration, temperature, pH, and salt ion concentration [82–84]. Such non-physiological conditions drive RBPs and associated macromolecules into hydrogel droplets with compromised dynamics, and even pathological crystal-like assemblies. Consistently, molecular crowding agents often lead to solid-like states of protein in vitro, referred to as protein aggregates [85].

Protein aggregation is the key feature of various NDDs, but the precise molecular mechanisms triggering aggregation are still largely unknown. Recent studies on SG assembly and disassembly through phase separation have shed light on the mechanisms of the transition between soluble and aggregated states of NDD-associated proteins (Table 1).

**Table 1:** SG assembly-related proteins in NDDs

	Names of proteins	Related NDDs	Pathogenic proteins affected	Interaction site or domain	Results	References
RNA binding protein	ANXA11	ALS		N-terminal-LCD-mutants (p.G38R, p. D40G)	The ANX domain missense variants alter SG disassembly	[86]
	ATXN2	ALS/SCA	–	ATXN2-IDR	ATXN2 localizes to SGs and promotes TDP-43 mis-localization. Decreasing ATXN2 prolongs lifespan and alleviates pathology in TDP-43 mice. ATXN2 IDR drives in vivo LLPS and in vivo RNP assembly	[87, 88]
	G3BP1	HD	HTT TDP-43	–	The superior frontal cortex of both R6/2 mice and human HD postmortem brain tissues were found with a notable increase in G3BP1 granules	[89]
		SCA	ATXN2/3	G3BP1-NTF2L	*G3BP1* overexpression decreased protein aggregation	[90]
		ALS-FTD	TDP-43	–	TDP-43 is colocalized with G3BP1-positive SGs, and TDP-43 can regulate the stability of *G3BP1* mRNA	[47, 91]
	hnRNPA1/A2/B1	ALS	TDP-43 FUS	hnRNPA2-D290 V hnRNPA1-D262 V hnRNPA1-D262 N hnRNPA1-P288 A (LCD-mutants)	Mutations in prion-like domains in hnRNPA2B1 and hnRNPA1 promote phase separation and regulate SG dynamics	[92, 93]
	hnRNPA2/B1	AD	Tau	–	Tau oligomerization induces striking cytoplasmic translocation of m6A to co-localize with HnRNPA2/B1 and oligo Tau	[94]
	FUS	ALS	–	FUS-R521C; FUS-P525L; arginine-rich RGG3 domain; FUS^p−Y526^	FUS mislocalization into cytoplasmic SGs	[72, 95–97]
	PABP/DDX6	AD	Tau	–	They are insoluble and colocalized with phosphorylated tau pathology	[98, 99]
	PABP	ALS	TDP-43	–	PABP modulates TDP-43 toxicity. Cytoplasmic PABP is mis-localized in spinal cord motor neurons	[100]
	TAF15	ALS	–	M368T, G391E, R408C, G473E	TAF15 variants showed cytoplasmic puncta formation in spinal cord neurons, and TAF15 can mislocalize into cytoplasmic SGs under cell stress	[101, 102]
	TDP-43	ALS	–	TDP-43 glycine-rich LCD at the C-terminal	TDP-43 as a component of neuronal RNP transport particles, exhibits liquid-like properties. The LCD also mediates LLPS and recruitment of TDP-43 to SGs	[103]
	TIA1	ALS-FTD	TDP-43	TIA1 LCD-P362L, E384K, A381T	*TIA1* mutations promote TDP-43 phase separation and alter SG dynamics, and make TDP-43 insoluble	[104, 105]
		AD	Tau	TIA1 interacts with Tau’s microtubule-binding domain	Tau phase separation is accelerated and the production of hazardous oligomeric tau is controlled by direct contact with TIA1	[82]
Other related protein	C9orf72	ALS-FTD	–	Hexanucleotide repeat expansions (G4C2)	Hexanucleotide repeat expansions lead to the abnormal accumulation of RNA lesions and the spontaneous formation of SGs. C9orf72 affects the clearance of SGs through interaction with eIF2α as well as autophagy receptor P62. Finally, abnormal accumulation of TDP-43 inclusion bodies is induced	[106–109]
	Karyopherin-β1/β2	ALS-FTD	FUS, TDP-43, hnRNPA1/A2	PrLDs	Karyopherin-β1/β2 activates PY-NLSs to prevent and reverse the fibrillation of TDP-43, FUS, TAF15, EWSR1, hnRNPA1, and hnRNPA2, and inhibits RBP recruitment to SGs	[110]
	SOD1	ALS	–	SOD1-L144F SOD1-G93A SOD1-A4V	Mutant SOD1 delays the formation of G3BP1- and TIA1-positive SGs by interacting with G3BP1 in an RNA-independent manner	[111]
			–	SOD1-G93A	TIA1 shows increased mis-localization and the interaction between SOD1 and TIA1 increases with disease progression and severity	[112]
	STAU1	SCA	ATXN2	–	STAU1 interacts with ATXN2 and regulates SG formation	[113]
	Ubiquitin2	ALS	–	–	Ubiquitin2 co-localizes with SG component proteins G3BP1, TIA1, ATXN2, and PABPC1. Ubiquitin2 mutation affects the assembly of SGs by regulating TIA1	[40]
	USP10	ALS	TDP-43	–	By boosting clearance of SGs and aggregate formations, USP10 prevents formation of aberrant TDP-43/TDP-35 aggregates in SG in neuronal cells	[81]
		AD	Tau	USP10 (1–274) USP10 (275–798)	USP10 overexpression induces TIA1/Tau/USP10-triple-positive SGs	[114]
	VCP	ALS/AD/FTD	–	–	Reduced VCP impairs SG assembly and disassembly	[115, 116]
Eukaryotic translation initiation factor	eIF2α	NDDs	–	–	eIF2α phosphorylation inhibits translation initiation complex formation and promotes SG formation	[22]
		AD	Tau	–	eIF2α is co-localized with phosphorylated tau	[98]
		ALS	TDP-43		SGs and translation-inhibiting eIF2α phosphorylation become abnormally upregulated on TDP-43 toxicity in *Drosophila*	[100]
	eIF3η	ALS	–	–	As a eukaryotic translation initiation factor as well as a marker of SGs, it can localize to mislocalized pathogenic proteins	[96]

AD, Alzheimer's disease; ALS, amyotrophic lateral sclerosis; ANXA11, annexin A11; ATXNT2, ataxin-2; C9orf72, Open Reading Frame 72 on chromosome 9; DDX6, DEAD-box helicase 6; eIF2α, eukaryotic translation initiation factor 2α; eIF3η, eukaryotic translation initiation factor 3η;EWSR1, Ewing’s sarcoma protein R1; FTD, frontotemporal dementia; FUS, fused in sarcoma; G3BP1, Ras-GAP SH3-domain-binding protein 1; HD, Huntington’s disease; hnRNPA1, heterogeneous nuclear ribonucleoprotein A1; hnRNPA2/B1, heterogeneous nuclear ribonucleoprotein; HTT, huntingtin protein; IDR, intrinsically disordered region; LCD, low complexity domain; m^6^A, N6-methyladenosine; MTBD, microtubule binding domain; NDDs, neurodegenerative diseases; NTF2L, nuclear transport factor 2-like; PABP, poly(A)-binding protein; PrLDs, Prion-like Domains; PY-NLS, PY-Nuclear localization signals; RGG, arginine‐glycine‐glycine repeat; RBPs, RNA binding proteins; SCA, spinocerebellar ataxia; SGs, stress granules; SOD1, superoxide dismutase 1; TDP-35, TAR DNA binding protein 35; TDP-43, TAR DNA binding protein 43; TAF15, TATA-box binding protein associated factor 15; tau-P2, proline-rich region P2; TIA1, T cell-restricted intracellular antigen-1; USP10, ubiquitin-specific peptidase 10; VCP, valosin-containing protein

### SGs in ALS and FTD

FTD and ALS are considered part of a spectrum due to their overlapping clinical features [106, 117]. TDP-43 and FUS pathologies are typical hallmarks of both ALS and FTD, but how these proteins start to aggregate remains unclear. Emerging evidence demonstrates that LLPS is a crucial molecular mechanism underlying ALS and FTD. Several genes encoding RBPs associated with LLPS, including *TDP-43*, *FUS*, *ATNX2*, *HNRNPA1*, and *TIA1*, are risk factors for both diseases [21, 87, 96, 104]. These RBPs can condense into SGs under stress and may form amyloid-like fibrillar aggregates [21].

Cytoplasmic TDP-43 aggregates are the primary neuropathological marker of ALS [118]. TDP-43 can mislocalize to SGs and then convert into cytoplasmic aggregates under stress [81]. TDP-43 proteinopathy is related to a deficiency in its nucleocytoplasmic transport. KPNB1 (Karyopherin-β), member of the nuclear import receptor family, can reverse abnormal phase transitions of Nup62 and TDP-43, inhibiting TDP-43 proteinopathy [118, 119]. Alternatively, in SH-SY5Y cells, exposure to fragmented TDP-43 or FUS fibrils leads to the formation of long-lived liquid droplets of cytosolic TDP-43 independent of SGs. Similarly, low-concentration SA treatment induces cytoplasmic TDP-43-containing particles [120]. In addition, RNA depletion promotes the formation of insoluble TDP-43 outside SGs compared with RNA-containing SGs [121]. The *TIA1* P362L mutation is a risk factor that delays SG disassembly and promotes the accumulation of non-dynamic, TDP-43-containing SGs, resulting in TDP-43 aggregation [104].

Hexanucleotide repeat expansions in the *C9orf72* gene are also related to FTD and ALS [122]. C9orf72 is strongly co-localized with the well-known SG markers G3BP1 and Hu-antigen R (HuR) under dithiothreitol treatment or heat shock [105, 123]. Ablation of C9orf72 completely abolishes SG assembly and accelerates cell death, while C9orf72 overexpression leads to spontaneous SG formation [106]. These findings suggest that phase transition-induced changes in SG dynamics may play a crucial role in the development of ALS and FTD.

### SGs in AD

AD is the most common form of dementia, primarily affecting memory in older adults [119]. The tau protein is the predominant component of intracellular neurofibrillary tangles, a pathological hallmark of AD. Tau can undergo LLPS, which facilitates tau amyloid aggregation. Intracellular phase separation of tau induces formation of subcellular foci of high local concentration of tau in neurons, which may lead to tau aggregation in the context of aberrant phosphorylation or mutations [124]. Tau droplet formation is enhanced in vitro under crowded conditions and can be further enhanced by the P301L mutation associated with inherited tauopathy [125]. LLPS regulates tau misfolding and drives its oligomerization [124, 126], initiating tau aggregation [124].

SGs produced through LLPS may be important in AD. Inhibiting SG assembly has been shown to alleviate AD-like pathology in mice [126]. Knockout of *TIA1* reduces SG formation, inhibits tau misfolding, and alleviates toxicity in primary hippocampal neurons [127]. LLPS of tau, driven by tau interactions with RNA and TIA1, can generate tau oligomers, emphasizing the importance of LLPS and TIA1 for tau pathology [82]. Interestingly, a decrease in SG assembly is associated with alleviation of tau pathology, suggesting that reducing SG assembly could inhibit AD progression [128]. However, other studies have shown inconsistent results. For instance, G3BP2, a major SG component, binds to the microtubule-binding region (MTBR) of tau through its NTF2 domain, inhibiting tau aggregation by masking MTBR. Loss of G3BP2 exacerbates tau pathology in primary human neurons and brain organoids [129]. These findings underscore the complex roles of SG and highlight the need for further studies on the functions of proteins like G3BP2 and TIA1. The role of LLPS-mediated SG assembly in AD remains controversial. Further studies on the mechanisms of LLPS and the SG dynamics in Aβ and tau aggregation are needed to advance the understanding of AD pathology.

### SGs in PD

PD is the second most common neurodegenerative disease after AD. It is characterized by progressive loss of dopaminergic neurons in the substantia nigra of the midbrain and intracellular accumulation of protein inclusions known as Lewy bodies (LBs) and Lewy neurites. The main protein component of the inclusions is α-synuclein (α-syn) [7], which is natively an unstructured protein that can aberrantly self-assemble into aggregates [130]. α-Syn aggregation is associated with PD pathogenesis [131], although the underlying mechanisms remain obscure.

Recent studies have shown that α-syn undergoes LLPS, which may precede its aggregation [83, 132]. In vitro studies showed that low pH, familial PD-associated mutations, and phosphomimetic substitution, factors known to aggravate α-syn aggregation, can also facilitate α-syn LLPS [133]. Similar results were observed in HeLa cells, where α-syn droplets evolve into perinuclear aggresomes [133]. These results suggest that α-syn can be concentrated in condensates, promoting amyloid formation through LLPS [134]. C-terminal truncation of α-syn, which is significantly increased in the brains of PD patients, regulates α-syn LLPS through electrostatic interactions [134]. Furthermore, C-terminally truncated α-syn can be recruited into wild-type (WT) α-syn droplets, accelerating WT α-syn aggregation through LLPS [132]. Therefore, LLPS-mediated α-syn self-assembly may be highly relevant in PD pathogenesis.

Neurotoxins such as rotenone, paraquat, MPTP (1-methyl-4-phenyl-1,2,3,6-tetrahydropyridine), and 6-OHDA (6-hydroxydopamine) are commonly used to establish cell and animal models of Parkinsonism. These toxins impair mitochondrial function, reducing ATP production [135], which can induce SG assembly due to ATP deficiency [18]. In this context, the dynamics of SGs may be altered when animals are treated with these toxins, leading to chronic stress that promotes α-syn aggregation. Therefore, investigating the pathological mechanisms mediated by SGs in toxin models can provide valuable insights into PD pathogenesis.

### SGs in Huntington’s disease (HD)

HD is a neurodegenerative disease characterized by neuropsychiatric symptoms, progressive cognitive impairment, and movement impairments. HD is caused by a dominantly inherited CAG trinucleotide repeat expansion in the huntingtin gene (*HTT*) [136]. Recent studies have revealed an association of SG assembly with HTT aggregation [89, 137]. Increased G3BP1-positive SGs have been observed in the cortex and hippocampus of R6/2 transgenic mice, a commonly used HD model, and in the prefrontal cortex of HD patients, suggesting an interplay between G3BP1 and HTT aggregation [89]. Outside SGs, HTT interacts with G3BP1. The recruitment of G3BP1 into SGs under stress conditions reduces its interaction with HTT, promoting mutant HTT aggregation in striatal neurons differentiated from patient-derived induced pluripotent stem cells. Besides, G3BP1 deficiency accelerates polyQ-expanded aggregation and toxicity in the neurons of HD *C. elegans* model [137].

The delicate balance of G3BP1 localization within and outside SGs appears to be crucial for modulating HTT aggregation dynamics, and is a potential therapeutic target for HD. Compared to ALS, FTD, and AD, studies on SG dynamics in HD are relatively rare, and there is a lack of sufficient evidence to fully understand the roles of SGs. Additional studies are required to determine whether SGs function as a protective ‘guardian’ or a detrimental ‘entity’ in HD pathogenesis through LLPS.

### SGs in spinocerebellar ataxias (SCAs)

SCAs are a large group of autosomal-dominant NDDs characterized by progressive cerebellar degeneration combined with brain stem atrophy, with complex molecular mechanisms in the pathogenesis [138]. Ataxin 2 (ATXN2) aggregates are a pathological feature in SCA2 brains [139]. Staufen 1 (STAU1) is abundant under multiple stressors and interacts with ATXN2 [140]. STAU1 levels are significantly elevated in SCA2 patient cells and animal models [141]. STAU1 is critical in SG assembly and disassembly [113]. STAU1 knockdown facilitates SG formation under stress conditions, while its overexpression impairs SG assembly [113].

Overexpression of *G3BP1* in Neuro2a cells decreases ATXN2 and ATXN3 aggregation, while silencing *G3bp1* in the mouse brain increases aggregation of human-expanded ATXN2 and ATXN3, suggesting a protective effect of G3BP1 against protein aggregation [90]. G3BP1 and its paralog G3BP2 are major components of SGs. However, SA-induced SG assembly reduces the overall protein translation, but does not affect the expression of ATXN2 and ATXN3 proteins or their aggregation. The relationship of G3BP2 with SG assembly was not explored in the study [90]. Although there is no direct evidence linking SG dynamics to SCA, multiple SCA risk genes have been revealed to regulate the process of LLPS or SG formation [90, 141]. These findings suggest a new research direction for exploring SCA pathogenesis mechanisms.

### SGs in prion disease

Prion diseases are rare neurodegenerative disorders characterized by the infectious conversion of normal cellular prion protein (PrP^C^) into its misfolded, disease-associated form PrP^Sc^ [142]. This conversion, which involves a structural shift from an α-helical to a β-sheet-rich conformation, propagates the disease by templating further PrP^C^ misfolding [142]. PrP has the property of LLPS, which is modulated by the interactions between PrP and nucleic acids [143, 144]. Nucleic acids not only influence the LLPS of PrP [143], but may also impact the pathological conversion of PrP by affecting its local concentration and aggregation propensity. In addition, PrP influences eIF2α regulation, as the presence of the misfolded, aggregating conformer PrP^sc^, correlates with enhanced eIF2α phosphorylation in prion-infected mice [145]. eIF2α dephosphorylation is neuroprotective in these mice [145], reinforcing this connection. Recently, the cellular PrP, PrP^C^, was shown to localize in TIA1-positive SGs after AS-induced stress in HeLa cells [146]. The cytoplasmic enrichment of PrP coincided with an alteration in its binding partners, which are predominantly related to RNA localization and processing [146].

Based on these findings, we can hypothesize that the elevated levels of p-eIF2α observed in prion-induced neurodegeneration may lead to the formation of PrP-containing SGs. This could represent a cellular defense strategy to mitigate the toxic effects of accumulating PrP^Sc^. However, the formation of PrP^Sc^ aggresomes disrupts this defense by preventing SG assembly in Neuro2a, BE(2)M17, and SK-N-SH neuroblastoma cells, as well as in HeLa cells under AS-induced stress [147]. The dysregulation of these stress responses could in turn enhance prion disease pathology by stabilizing PrP^Sc^ and facilitating its spread.

## Critical domains of RBPs for SG assembly in NDDs

RBPs contain RNA-binding domains and regulate RNA metabolism and function. They also regulate LLPS during stress, contributing to SG assembly. The various sequence domains in RBPs, including RNA recognition motifs (RRMs), low complexity domains (LCDs), nuclear transport factor 2-like (NTF2L), IDR (intrinsically disordered regions), Arginine (R)-Glycine (G) (RG) motif, and Arginine (R)-Glycine (G)-Glycine (G) (RGG) motif (Table 2), play significant roles in regulating LLPS, especially for SGs. In this section, we will summarize the roles of RRMs, LCD and NTF2L domains in the pathologies of NDDs.

**Table 2:** Proteins and domains that facilitate LLPS in NDDs

Names of proteins	RNA-binding domain			LLPS-promoting domains				References
ATXN2	LSm	LSmAD		Poly Q (548–571)	Poly Q (616–656)	cIDR (900–1084)	–	[88, 148]
FUS	RRM	–	–	PrLD	RGG (371–422)	RGG (453–501)	PY-NLS (501–526)	[149]
G3BP1/2	RRM	–	–	NTF2L	Gly-rich	–	RGG	[34, 90]
hnRNPA1	RRM1	RRM2	–	LCD	–	–	–	[92, 150]
hnRNPA2/B2	RRM1	RRM2	–	LCD				[150]
Tau	–	–	–	PRD (P1, P2)	MTBD (R1, R2, R3, R4)	–	–	[82, 151]
TDP-43	RRM1	RRM2	–	LCD	–	–	–	[149]
TIA1	RRM1	RRM2	RRM3	LCD	–	–	–	[104]

ATXNT2, ataxin-2; FUS, fused in sarcoma; cIDR, C-terminal intrinsically disordered region; G3BP1/2, Ras-GAP SH3-domain-binding protein 1/2; Gly-rich, Glycine rich region; hnRNPA1, heterogeneous nuclear ribonucleoprotein A1; hnRNPA2/B1, heterogeneous nuclear ribonucleoprotein; LCD, low complexity domain; LSm, like-Sm; LSmAD, like-Sm associated domain; MTBD, microtubule-binding domain; NTF2L, nuclear transport factor 2-like; PolyQ, Polyglutamine; PRD (P1/2), Proline-rich domain 1/2; PrLD, Prion-like Domain; PY-NLS, PY-Nuclear localization signals; RGG, arginine‐glycine‐glycine repeat; RRM, RNA recognition motif; TDP-43, TAR DNA-binding protein 43; TIA1, T cell-restricted intracellular antigen-1

### RRMs

RRMs are typical RNA-binding domains and are among the most abundant and extensively studied domains in RBPs [152]. Most RBPs contain one or more RRM domains, which interact with various nucleic acids and proteins to regulate RNA metabolism and gene expression [153]. In ALS, tethered TDP-43 RRM1-RRM2 is prone to aggregation and fibrillation [153]. The FUS RRM domain, characterized by high thermodynamics and conformational dynamics, can spontaneously self-assemble into amyloid fibrils [154], suggesting a pivotal role in transiting FUS from the reversible and physiological state to the irreversible pathological states.

Furthermore, deletion of the FUS RRM domain facilitates LLPS through the loss of inhibitory intramolecular interactions between the RRM and the RGG domains [155]. This is similar to the finding that RNA binding to the TDP-43 RRM domains blocks neurotoxic phase transitions of TDP-43 by inhibiting intramolecular interactions [121]. Similar results were observed in the heterogeneous nuclear ribonucleoprotein A1 (hnRNPA1) protein, in which multivalency interactions between RNA and RBPs contribute to LLPS [21]. In addition, PTMs of RRM domains associated with SG formation can influence protein aggregation. For example, acetylation of TDP-43 RRMs promotes protein aggregation [156]. These findings suggest that RRMs may be crucial in regulating LLPS processes.

### LCDs

LCDs serve as intrinsic molecular drivers of LLPS through their sequence-encoded biophysical properties [21]. Multiple RBPs harbor LCDs enriched with amino acid residues, particularly glycine, tyrosine, and serine, which enhance structural flexibility and disorder [157]. Glycine promotes droplet fluidity, while glutamine enhances droplet hardening and decreases droplet dynamics [157]. The LCD of hnRNPA1 mediates LLPS and incorporates it into SGs [21]. In ALS-FUS, mutations in the LCD domain may accelerate FUS LLPS into less dynamic or irreversible fibrils, contributing to pathological protein aggregates in patient cells [158]. Similar results were observed for the LCD of TATA-binding protein-associated factor 15 (TAF15) and hnRNPH1, in which Y-to-S mutations in G/S-Y-G/S inhibit the liquid droplet assembly [159].

In addition to FUS and TDP-43, mutations in the LCD of other RBPs related to FTD and ALS, such as EWSR1 (Ewing sarcoma breakpoint region 1), TIA1, TAF15, Ataxin2, hnRNPA1, and hnRNPA2B1, have also been reported to interfere with SG assembly [150]. Collectively, these genetic and mechanistic findings demonstrate that, LCDs act as central molecular scaffolds governing the primary determinant of cell phase separation and regulating SG dynamics. For instance, the LCD of TIA1 is essential for SG assembly, and ALS-associated proline-to-leucine mutations in TIA1 lead to abnormal SG kinetics and acceleration of TIA1 fibrillization [160]. hnRNPA1 with LCD mutation such as D262V, showed delayed SG disassembly two hours after the removal of SA, as observed in SH-SY5Y cells [161]. Taken together, these studies indicate that LCDs of RBPs, especially those associated with LLPS, play pivotal roles in regulating SG assembly and disassembly. Aberrant phase separation can significantly impact SG dynamics, influencing NDD pathogenesis.

### NTF2L domain

The NTF2L domain is a conserved protein domain found in RBPs [74]. G3BP1, the core component of SGs, harbors an NTF2L domain essential for protein translocation through the nuclear pore complex [74]. In cell models, overexpression of G3BP1 leads to decreased ATXN2 and ATXN3 aggregation, while NTF2L deletion increases aggregation [90]. In addition, the NTF2L domain in the N-terminus of G3BP2 can interact with tau in its R2–R3 repeats and significantly decrease tau aggregation [129]. Although these studies did not evaluate the assembly of SGs, current evidence suggests that NTF2L will impact the localization and concentration of RBPs, thereby affecting SG formation. Moreover, a previous study in COS cells showed that the NTF2L domain mediates G3BP recruitment to SGs [162]. In addition, Caprin-1 enhances the formation of SGs via its RNA-binding domain at the C-terminal. In contrast, the NTF2L domain of G3BP1 interacts with the G3BP1-interacting motif of Carprin-1, inhibiting G3BP1 phase separation [163]. These findings highlight the significant role of the NTF2L domain in SG formation and dynamics, which warrants further investigation into its contribution to NDD pathogenesis.

## SG assembly-disassembly is a double-edged sword for NDDs

SG assembly and disassembly has a complex and multifaceted role in cellular stress response. During age-associated alterations such as oxidative stress and inflammation, RNAs bind to RBPs to form complexes, leading to SG assembly with defensive functions [164]. At physiological states, SG assembly can reduce cellular energy demands and maintain protein homeostasis [165]. SGs immediately disassemble after stress recovery, which is essential for restoring normal cellular metabolism [166].

However, during aging, SGs can have detrimental effects. Aging is the most important risk factor for pathological protein aggregation in NDDs [167]. Since neurons are long-lived cells constantly exposed to mild microenvironmental stress—less intense than the acute stresses applied in cell line studies (in vitro)—the formation of large SGs may not occur [164]. During this chronic stimulation, SGs gradually transit from a small reversible state to a larger irreversible one. These irreversible SGs may act as seeds that trigger the formation of a nucleus. This nucleus slowly transits from a liquid phase to a gel phase and eventually to a solid phase. This process ultimately promotes the buildup of toxic proteins in neurons, contributing to the accumulation of pathological proteins in age-related neurological disorders [120, 128].

Initially produced through LLPS as a protective mechanism in eukaryotic cells, the role of SGs in NDDs still requires further investigation. Numerous in vitro experiments have demonstrated that SGs are key players in enhancing the aggregation of proteins associated with NDDs [8, 49]. Evidence from cell models suggests that soluble RBPs may be the “initiated nidus” for forming larger pathological aggregates that bind with or without pathological proteins associated with NDDs [99]. RBPs, such as G3BP1 and TIA1, are the primary components of SGs and can aggregate into noticeable granules that eventually evolve into SGs [99]. SGs may serve as a pivotal trigger for pathological protein aggregation in NDDs when SG dynamics are imbalanced. Overexpression of RBPs decreases ATXN2 and ATXN3 aggregation while silencing G3BP1 increases human ATXN2 and ATXN3 aggregation during aging. This emerging evidence suggests a potential pathomechanism involving dysregulated SG homeostasis, characterized by upregulated condensate nucleation concomitant with compromised disassembly kinetics. This functional imbalance manifests as persistent SG accumulation due to attenuated clearance machinery, ultimately driving the transition from dynamic liquid droplets to pathogenic solid aggregates. The imbalance in SG dynamics under the pathological conditions of NDDs remains a topic of debate.

However, SGs may counteract neuronal damage and inhibit protein aggregation at the early stages of NDDs. In primary skin fibroblasts from ALS patients with TDP-43 A382T mutation, the number of SGs per cell and the percentage of cells that form SGs under SA stress are decreased compared to cells with wild-type TDP-43. Although there is no direct evidence for the association of reduced SG formation with pathological exacerbation in the TDP43 mutant cell line, what is clear is that the fibroblasts with A382T mutation have a defective SG response [168]. The SG assembly deficits have also been observed in aged primary neurons from ALS mice with TDP-43 M337V mutation, when exposed to heat shock [169]. Therefore, SG formation can be protective under certain conditions. However, prolonged or dysfunctional SG assembly and impaired SG disassembly can have deleterious effects on neuronal health. This double-edged sword nature of SGs underscores the need for a better understanding of their regulation and function in the processes of NDDs.

The complexity of the pathogenesis of NDDs poses tremendous challenges for developing therapeutic strategies. Traditional therapeutic approaches, such as neuroprotective agents, gene therapy, anti-inflammatory therapy [170], and surgical technologies, focus primarily on alleviating motor symptoms of NDDs without addressing the underlying disease mechanisms [171–174]. Current research emphasizes identifying key players and targeting them to delay the onset or progression of NDDs. Therefore, screening for early diagnostic biomarkers and treatment targets within the SG-interacting RBPs network may lead to the development of novel therapeutic strategies that target the root causes of NDDs.

## Conclusions

**Fig. 2:**
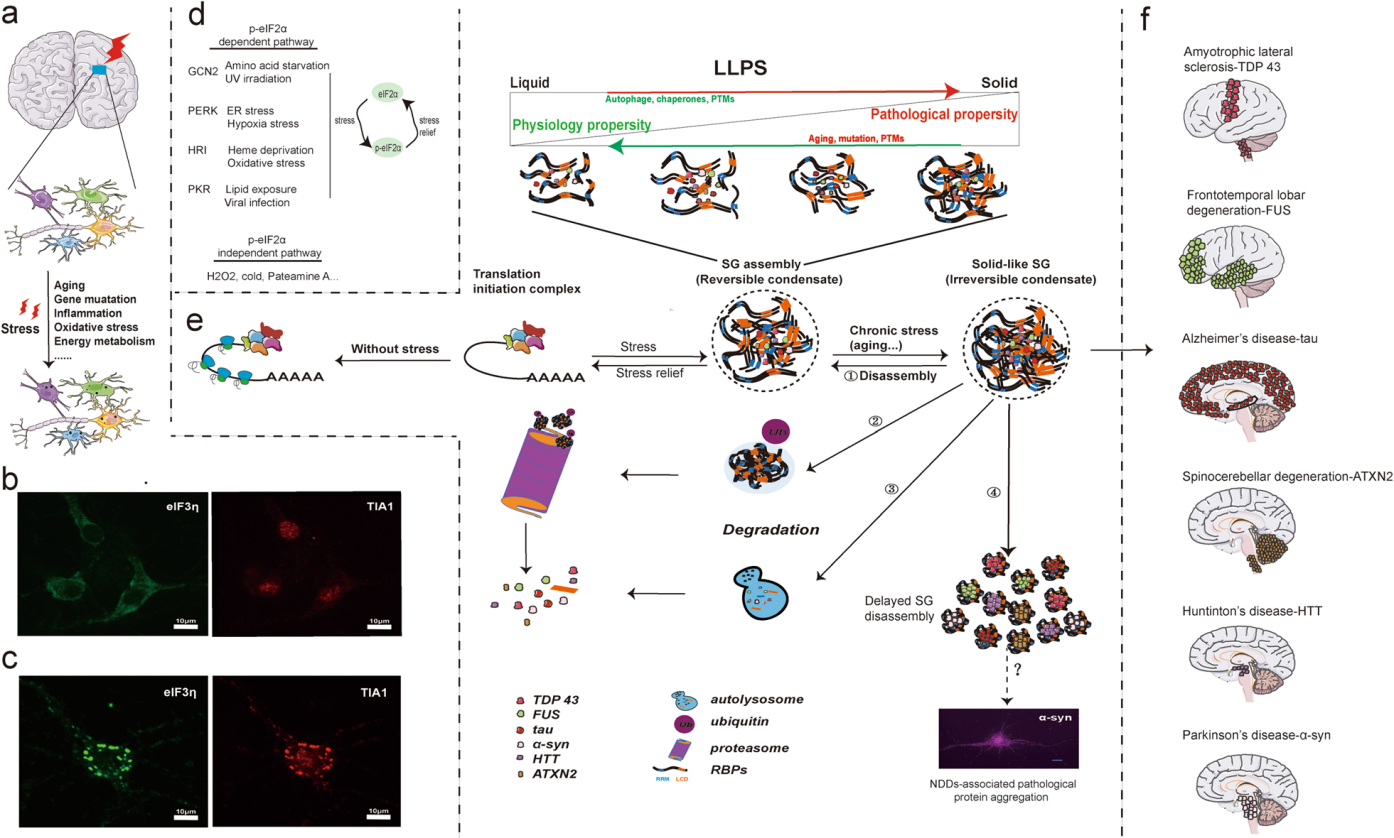
The potential contribution of SG assembly to NDDs through LLPS. **a** Neurons, astrocytes, microglia, and oligodendrocytes are the major cell components in the central nervous system. SG assembly occurs in the cytoplasm of these cells under environmental or endogenous stimuli. **b**, **c** Immunofluorescence images of SGs in the cytoplasm of primary mouse cortical neurons. Green, eIF3η (Santa Cruz Biotechnology, Dallas, TX; sc-37214); red, TIA1 (Abcam, Cambridge, United Kingdom, ab140595). Scale bar: 10 μm. Images were provided by our team member, Li-Hong Mao. **d** SG assembly can be induced by the p-eIF2α pathway that activates four kinases: GCN2, PERK, HRI, and PKR. SGs can also be induced independently of the p-eIF2α pathway under stress conditions such as H_2_O_2_, cold shock, and Pateamine A treatment. **e** SG assembly through LLPS. RBPs assemble into liquid-like phase-separated condensates with pathological proteins, through multivalent interactions with liquid droplets (dynamic), hydrogels (weakly dynamic), and amyloid-like fibrils (non-dynamic). ① Following stress relief, SGs transiently dissipate via the ubiquitin–proteasome system (UPS)- or autophagy-independent disassembly, and release the RBPs, mRNAs, and the proteins associated with NDDs. ②, ③ The SGs may also be cleared by the UPS- or autophagosome/lysosome-dependent disassembly. ④ The transient storage of these RBPs, mRNAs, and pathological proteins impairs SG disassembly, eventually resulting in pathological protein aggregation. The immunofluorescence image stained by our lab shows α-syn aggregates in primary mouse cortical neurons (in purple (Santa Cruz Biotechnology, Dallas, TX; sc-12767)). Scale bar: 10 μm. **f** Illustration of brain regions affected by pathology in different NDDs. When the process of SG assembly and disassembly is disrupted, SGs may be either directly or indirectly implicated in NDDs

Despite decades of research on the mechanisms of pathological protein aggregation and the development of therapeutic strategies to prevent this process, the initiation of pathological protein aggregates remains elusive. Dyshomeostasis of SG dynamics contributes to pathological protein aggregation. Recent studies suggest that LLPS increases protein concentrations [175], which can disrupt the dynamic homeostasis of SG assembly and disassembly, creating a feedback loop that initiates misfolded protein aggregation in NDDs. The dynamic homeostasis of SGs promotes the transition of pathological proteins from soluble to insoluble states, resulting in pathological protein aggregation (Fig. 2). Starting from the "primitive culprit" mediated by LLPS, the dynamic imbalance of SGs may drive the accumulation of pathogenic proteins in NDDs [104, 176].

Modulation of RBP expression is critical for maintaining the dynamic equilibrium of protein solutions. For instance, reducing TIA1 level offers protection against tauopathy, while mutant FUS drives early FUS ALS neurodegeneration [177]. Knockdown of G3BP1 has been linked to increased mutant HTT aggregation [137], whereas reducing G3BP2 levels leads to tau aggregation [129]. Conversely, overexpression of G3BP1 decreases ATXN2 and ATXN3 protein aggregation and preserves neuronal cell function [90]. Mitigation and exacerbation of protein aggregation states by RBP dysregulation perturbs SG homeostasis. Investigating the roles of RBPs in regulating SG assembly and disassembly as well as the roles of SGs at different stages of NDDs may advance our understanding of NDD pathogenesis and lead to discovery of potential therapeutic options.

Although SGs are transient membraneless structures formed in response to stress, the role of SGs in the long-term pathogenesis of NDDs remains significant for further exploration. Our current understanding of the contribution of SGs to NDDs is still limited. Further research is needed to investigate the involvement of SGs in NDDs. It is also important to determine whether drugs targeting NDD or factors aggravating NDD pathogenesis can stimulate SG formation, whether the major components of SGs are different under various conditions, and the roles of protein components within SGs. Addressing these questions may significantly deepen our understanding of NDD pathogenesis and uncover potential therapeutic options.

## Data Availability

Not applicable.

## Abbreviations

α-Syn: α-Synuclein
AD: Alzheimer’s disease
ALS: Amyotrophic lateral sclerosis
ATP: Adenosine triphosphate
ATXN2: Ataxin 2
DDX6: DEAD-box helicase 6
FTD: Frontotemporal dementia
FUS: Fuse in sarcoma
G3BP: RasGAPSH3-binding protein
HD: Huntington’s disease
hnRNPH1: Heterogeneous nuclear ribonucleoprotein H1
Hsp: Heat shock protein
HSPB1: Heat shock protein family B (small) member 1
LBs: Lewy bodies
LCD: Low complexity domain
LLPS: Liquid–liquid phase separation
MTBR: Microtubule-binding region
m6A: N6-methyladenosine
NDD: Neurodegenerative disease
NTF2L: Nuclear transport factor 2-like
PD: Parkinson’s disease
RBP: RNA-binding protein
RRM: RNA recognition motif
SA: Sodium arsenite
SCA: Spinocerebellar ataxia
SGs: Stress granules
TAF15: TATA-binding protein-associated factor 15
TIA1: T-cell restricted intracellular antigen 1
TRIM21: Tripartite motif containing 21
UPS: Ubiquitin-proteasome system
